# Supplementary material for: Can green funds improve corporate environmental, social, and governance performance? Evidence from Chinese-listed companies
Source: PLoS One. 2024 Mar 28;19(3):e0301395. doi: 10.1371/journal.pone.0301395 (PMC10977774; doi:10.1371/journal.pone.0301395)
Supplement: S1 File — (DOCX) [file pone.0301395.s001.docx]

**Supporting information**

**S1. Variable definitions**

Table A: Variable definitions

|  | Main variables | Definition | Data sources |
| --- | --- | --- | --- |
| Dependent variable | *ESG* | Huazheng ESG Rating, including three dimensions: environment (*E_score*), social responsibility (*S_score*) and governance (*G_score*) | Wind |
| Independent variable | *lngreen* | The log number of green funds that the company received in the current year plus 1 | CSMAR |
| Control variables | *Size* | The log of the total assets of the company at the end of the year | CSMAR |
|  | *Lev* | The ratio of total liabilities to total assets of the company. |  |
|  | *ROA* | The ratio of net profit to total assets of the company |  |
|  | *FirmAge* | The log of the difference between the current year and the year of establishment of the company |  |
|  | *Cashflow* | The ratio of net cash flows from operating activities to total assets of the company |  |
|  | *TOP1* | The proportion of equity held by the largest shareholder of the company in the current year |  |
|  | *Indep* | The ratio of the number of independent directors to the total number of directors in the company |  |
|  | *Board* | The log number of directors in the company's board. |  |
| Alternative explanatory variable | *green* | A dummy variable indicating the presence (1) or absence (0) of green funds in the company during the current year. | CSMAR |
|  | *greenratio* | The proportion of the market value of shares held by green funds to the net value of the company. | CSMAR |
| Alternative dependent variable | *ESG_pengbo* | Bloomberg's released corporate ESG scores (available since 2011) | Bloomberg |
| Heterogeneous grouping variables | *media* | The log frequency of company names in the titles of print and online news articles | CNRDS |
|  | *analyst* | The log number of analysts tracking the company in the current year | CNRDS |
|  | *Public* | The log of the annual average Baidu search volume for the terms "environmental pollution" and "haze" in the city where the company is located plus one. | www.baidu.com |
|  | *regulation* | The log frequency of terms related to environmental regulation in the government work reports | Prefectural-level Municipal Government Information Disclosure Website |
|  | *air pollution* | The log of the average PM2.5 concentration | NASA |
|  | *market* | The marketization index proposed by Fan Gang | CNRDS |
|  | *SA* | SA index, a measure of corporate financing constraints | CSMAR |
|  | *FC* | FC index, a measure of corporate financing constraints | CSMAR |
| Mechanism variables | *Cost* | Proportion of total interest expenses plus fees and other financial expenses to total year-end liabilities | CSMAR |
|  | *MPEG* | Corporate cost of equity financing calculated by MPGE model | CSMAR |
|  | *Interfund* | Ratio of net cash flow generated from operating activities to lagged total assets | CSMAR |
|  | *ATO* | Total asset turnover ratio | CSMAR |
|  | *Myopia* | Ratio of the total word frequency representing short-sighted behavior to the total word frequency multiplied by 100 | Corporate Social Responsibility Report from the Wingo Platform |
|  | *lnIC* | The log of the internal control index | DiBo database |
|  | *lnGreTotal* | The log number of total green patents applied by the company in the current year plus one | CNRDS |
|  | *lnGreInvia* | The log number of green invention patents applied by the company in the current year plus one | CNRDS |
|  | *lnGreUmia* | The log number of green utility model patents applied by the company in the current year plus one | CNRDS |
| Further analysis of variables | *TFP_LP* | Total Factor Productivity (TFP) calculated using the LP method | CSMAR |
|  | *TFP_OP* | Total Factor Productivity (TFP) calculated using the OP method | CSMAR |
|  | *TobinQ* | Tobin's Q value for the company | CSMAR |
|  | *PB* | Price-to-Book (P/B) ratio for the company | CSMAR |
| Other variables | *EPC* | Environmental protection concept | CSMAR |
|  | *EG* | Environmental protection goal |  |
|  | *EMS* | Environmental management system |  |
|  | *EDT* | Environmental protection education and training |  |
|  | *EA* | Environmental protection unique action |  |
|  | *EEM* | Environmental emergency mechanism |  |
|  | *EHA* | Environmental protection honors or rewards |  |
|  | *TS* | Three simultaneous systems |  |
|  | *IV* | the interaction term consisting of whether the fund manager has a green background and the average green fund shareholding proportion of other firms in the same industry and the same year (excluding the focal firm) | The official website of the fund company and CSMAR |
|  | *Indgreenfund* | The industry-average green fund shareholding proportion excluding the focal firm | CSMAR |
|  | *CPU* | The climate policy uncertainty index which reflects regulation pressure | World News and WISER information databases |
|  | *gws* | Greenwashing behavior according to Hu et al. (2023) | Firm's annual reports from the Wingo Platform, CSMAR and CNRDS databases. |
|  | *LCCP* | Low-carbon city pilot policy | Prefectural-level Municipal Government Information Disclosure Website |
|  | *Carbon* | Carbon market trading pilot policy |  |
|  | *GCP* | Green credit policy |  |

**S2. Green-related key words**

The inclusion of terms such as "environmental protection," "ecology," "green," "new energy development," "clean energy," "low-carbon," "sustainable," "energy efficiency," and "beautiful China" within the "investment objectives" and "investment scope" is indicative of green funds.
