# Supplementary material for: Can green funds improve corporate environmental, social, and governance performance? Evidence from Chinese-listed companies
Source: PLoS One. 2024 Mar 28;19(3):e0301395. doi: 10.1371/journal.pone.0301395 (PMC10977774; doi:10.1371/journal.pone.0301395)
Supplement: Attachment — Submitted filename: Response+to+Reviewers.docx [file pone.0301395.s003.docx]

**Reply to Reviewer 1**

Dear Professor,

We sincerely thank you for your helpful and constructive comments and suggestions. We have considered your advice very seriously and incorporated it into the revised paper. We believe the paper is much improved and sincerely thank you for the advice and encouragement. Thank you for your kind comments on this manuscript. This statement details the changes we made, and it is keyed to your report. For ease of reading, we have included the original comments in bold font, followed by our detailed response in highlighted blue font. The reply to the comments is listed as follows.

**While the paper provides valuable insights into the influence of green funds on ESG performance in Chinese A-share listed companies, there are a few areas where the authors could further develop and improve their work:**

**[Comment 1] Causality and endogeneity: The authors should address the issue of endogeneity in their analysis. Green funds may be attracted to companies with better ESG performance, rather than being the cause of improved performance. To strengthen the causal claims made in the paper, the authors could consider employing instrumental variable techniques or conducting a difference-in-differences analysis.**

**Response:**

*Thanks for your comments. As for the endogeneity test, we re-select a more exogenous instrumental variable for analysis. The revised manuscript is as follows:*

**4.4.1 IV regressions**

The enhancement of corporate ESG performance might not solely result from alleviating financial constraints and external monitoring prompted by green funds. It might be driven by the phenomenon that companies with better ESG performance attract more green funds. Furthermore, unobservable factors influence both ESG performance and green funds. The presence of reverse causality and omitted variables could lead to endogeneity issues in this study. Therefore, we employ the interaction term consisting of whether the fund manager has a green background and the average green fund shareholding proportion of other firms in the same industry and the same year (excluding the focal firm) as an instrumental variable (IV). Among them, the background information data of the fund manager is manually crawled from the official website of the fund company. If the enterprise is held by at least one fund with green background manager, the value is assigned to 1. The reason for this is that, on the one hand, the fund manager's green background influences the investment style and directs attention towards environment-friendly projects, consequently leading to investments in green funds. Therefore, this satisfies the relevance of IV^[1]^; on the other hand, the link between corporate ESG performance and the fund manager's previous green experience is fragile, satisfying the exogeneity of IV. This study employs a Two-Stage Least Squares (2SLS) approach to perform instrumental variable regression. The two-stage regression model is presented as Equations (2) and (3):

$$\ln green_{it}=\beta_{0}+\beta_{1}IV_{it}+\beta_{2}Controls_{it}+\varpi_{i}+\varpi_{t}+\varepsilon_{it}^{'}$$

$$ESG_{it}=\alpha_{0}^{'}+\alpha_{1}^{'}\widehat{\ln green_{it}}+\alpha_{2}^{'}Controls_{it}+\mu_{i}^{'}+\delta_{t}^{'}+\varepsilon_{it}^{' '}$$

The results are presented in Table 12. Column (1) represents the first-stage regression outcome, indicating that IV positively relates to the number of green funds entering the focal company for the current year. The result of KleibergenPaap LM statistics rejects the assumption that the instrumental variable is unrecognized. The results of Cragg Donald Wald F and Kleibergen-Paap rk Wald F statistics reject that there is a weak instrument problem in this study. All the statistics are greater than the critical value at the 1% level. Hence, this instrumental variable is effective. Column (2) presents that the impact of green funds on corporate ESG performance is significantly positive, validating the robustness of the baseline regression results.

**Table 12. IV estimations**

|  | (1) | (2) |
| --- | --- | --- |
|  | lngreen | ESG |
| *IV* | 11.9489^***^ |  |
|  | (1.5220) |  |
| *lngreen* |  | 0.4646^**^ |
|  |  | (0.2017) |
| Controls | Yes | Yes |
| Firm fixed effects | Yes | Yes |
| Year fixed effects | Yes | Yes |
| N | 29991 | 29991 |
| Kleibergen-Paap rk LM statistic | 52.906  61.635  109.482 | |
| Kleibergen-Paap rk Wald F statistic |  |  |
| Cragg-Donald Wald F statistic |  |  |

**[Comment 2]** **Generalizability: While the study focuses on Chinese A-share listed companies, it would be helpful to discuss the generalizability of the findings to other contexts. Are there any unique characteristics of the Chinese market that may limit the applicability of the results to other countries or regions? Discussing the external validity of the findings would enhance the broader relevance of the study.**

**Response:**

*Thank you for your insightful comments regarding the generalizability of our study. We appreciate your suggestion to discuss the external validity of our findings. Our results are applicable to other countries or regions.*

*We have made some supplements in the discussion and limitation section at the end of the article.* *The revised manuscript is as follows:*

Although this study only uses listed companies in China as the research sample, the conclusions are practically relevant for many countries with emerging economies, including China, for two main reasons. Firstly, most emerging economies share similar characteristics with China since their green financial systems are not yet fully developed and the concept of green sustainable development is yet to be promoted [75,76]. Therefore, exploring how to guide sustainable development with the support of green funds is an integral part of green financial reform. Secondly, literature about green funds in different regions also provides support for the applicability of our conclusions. Ma et al. (2023) [61] discussed the relationship among green fund concerns, corporate R&D investment and sustainable development. He et al. (2022) [69] and Jiang and Bai (2022) [77] analyzed the differential impact of institutional investors on corporate green innovation. In addition, Siemroth and Hornuf (2023) [78] found that investors prefer positive environmental impact and drive corporate transformation by investing in green projects. As for the performance of green funds in international markets, Goodell et al. (2022) [79] discovered that green funds are related to the development of financial technology. Ji et al. (2021) [76] revealed that the green funds of BRICS have performed better than other types of funds. Sangiorgi and Schopohl (2021) [80] and Chatnani (2018) [81] also found green investing funds in Europe and India have a better performance in stock price. Silva and Cortez (2016) [82] found that the performance of US and European green funds is higher in crisis periods. In general, our study is based on the above research and further focuses on the impact of green funds on the sustainable development of enterprises, rather than the single dimension of corporate environmental performance, stock price, or investment return considered in previous literature.

Our study extends the aforementioned research and delves deeper into examining green funds and corporate ESG performance. However, it is essential to note that this paper has yet to explicitly address the impact of small and medium-sized enterprises (SMEs), which remain pivotal to economic development in many emerging economies. Therefore, future research could be broadened to include SME data.

**[Comment 3]** **Counterarguments and limitations: It is important to acknowledge potential counterarguments and limitations of the study. For example, are there any potential negative effects or unintended consequences of green funds on ESG performance that should be considered? Addressing these points would provide a more balanced perspective and strengthen the overall argument.**

**Response:**

*Thank you for your insightful feedback. We appreciate your suggestion to address potential counterarguments and limitations in our study, and we agree that acknowledging unintended consequences is crucial for a more comprehensive understanding. In the regression analysis, we discuss unintended consequences from the following two aspects: Firstly, we further investigate the differentiated impact of green funds on three dimensions of E, S, and G. The revised manuscript is as follows:*

In addition, this study further analyses the differential impact of green funds on corporate environmental, social, and governance performance by splitting the ESG scores into three dimensions: E, S, and G. According to the regression results in Table 5, it is evident that green funds primarily enhance corporate environmental (*E_score*) and governance (*G_score*) performance. However, the effects of green funds on social responsibility performance (*S_score*) are relatively small. Therefore, green funds may be more inclined to focus on corporate environmental awareness and internal governance performance, and may not pay enough attention to promoting corporate social responsibility.

**Table 5. The impact of three dimensions**

|  | (1) | (2) | (3) |
| --- | --- | --- | --- |
|  | *E_score* | *S_score* | *G_score* |
| *lngreen* | 0.0655^***^ | 0.0209^*^ | 0.0822^***^ |
|  | (0.0108) | (0.0111) | (0.0145) |
| Controls | Yes | Yes | Yes |
| Firm fixed effects | Yes | Yes | Yes |
| Year fixed effects | Yes | Yes | Yes |
| N | 29991 | 29991 | 29991 |
| Adj. R^2^ | 0.5130 | 0.6275 | 0.4633 |

*Secondly, we explore and discuss any adverse impacts that may arise from the influence of green funds, considering both large and small enterprises. This implies a discussion of how different company sizes might experience varying effects from green funds. The specific ideas are mainly:*

**Table 18. Heterogeneous analysis: firm characteristics**

|  | (1) | (2) | (3) | (4) | (5) | (6) |
| --- | --- | --- | --- | --- | --- | --- |
|  | Ownership | | Pollution | | Size | |
|  | SOE | non-SOE | Low | High | Small | Large |
| *lngreen* | 0.0371** | 0.0853*** | 0.0557*** | 0.1103*** | 0.0115 | 0.0817*** |
|  | (0.0170) | (0.0140) | (0.0120) | (0.0243) | (0.0164) | (0.0143) |
| Controls | Yes | Yes | Yes | Yes | Yes | Yes |
| Firm fixed effects | Yes | Yes | Yes | Yes | Yes | Yes |
| Year fixed effects | Yes | Yes | Yes | Yes | Yes | Yes |
| N | 10892 | 19099 | 22812 | 7179 | 14995 | 14996 |
| Adj. R2 | 0.5423 | 0.5113 | 0.5254 | 0.4992 | 0.5267 | 0.5357 |
| Experience P-value | 0.000*** | | 0.000*** | | 0.000*** | |

The coefficient of *lngreen* is higher in the large enterprise group, while insignificant in the small enterprise group. Permutation tests reveal a significant difference between the two groups, indicating that green funds have a more substantial promoting effect on the ESG performance of larger enterprises. This finding implies that the influence of green funds is more substantial in larger corporations, potentially leading to a reinforcing effect that the stronger entities become stronger, and the weaker ones relatively weaker. Consequently, this may contribute to an amplification of differences among enterprises, creating environmental sustainability gaps between more and less resourceful entities.

*As for the limitation of our study, we discuss at the end of the passage:*

Our study extends the aforementioned research and delves deeper into examining green funds and corporate ESG performance. However, it is essential to note that this paper has yet to explicitly address the impact of small and medium-sized enterprises (SMEs), which remain pivotal to economic development in many emerging economies. Therefore, future research could be broadened to include SME data.

*These adjustments aim to explicitly address the potential negative effects, unintended consequences of green funds on ESG performance and the limitations of this study, providing a more balanced perspective and strengthening the overall argument of our study.*

**[Comment 4]** **Policy implications: While the authors provide some** **policy recommendations based on their findings, it would be helpful to elaborate on the practical implications of these recommendations. How feasible are these policy suggestions, and what challenges might arise in their implementation? Providing more detailed guidance for policymakers would enhance the practical relevance of the study.**

**Response:**

*Thanks for your comments.* *To ensure policy recommendations are more targeted and practical, we have rewritten this section from three perspectives: green financial system, external supervision, and green innovation. The revised manuscript is as follows:*

Firstly, the government should promote the construction of a comprehensive green financial system to facilitate the flow of green funds to enterprises and reduce the threshold for green financing. For example, it should offer tax reductions or exemptions to individual and institutional investors showing a strong willingness to invest in green funds. What’s more, the government can establish a dedicated fund to connect green funds with enterprises, and provide financial subsidies or low-interest loans for green projects to lower the cost of environmental protection financing for enterprises, which can incentivize more capital to flow into green funds and promote more investment in environmental protection and sustainable development, thereby increasing the positive impact on corporate ESG performance. To enhance the inclusivity of green funds, the government can introduce a range of financing policies that lower the funding threshold for small-scale enterprises. Simultaneously, it should encourage fund investors to focus on the growth potential of these smaller enterprises, so that green funds can better empower their development. Additionally, it is imperative to establish a specialized regulatory body tasked with overseeing the flow of green funds to avert the potential issue of "greenwashing." This regulatory department plays a crucial role in ensuring the legitimacy and authenticity of green investments, safeguarding the integrity of the overall green financial system.

Secondly, companies should recognize the supervisory effect exerted by external attention and ensure the efficient utilization of green funds. Ensuring the prudent use of green funds is paramount for maximizing their values. As crucial external stakeholders, green funds not only provide financial support for green development but also play a vital role in overseeing corporate performance. Consequently, companies should prioritize the strategic roles of external attention and ESG, actively engage in ESG practices, and enhance their capacities for sustainable development. Moreover, recognizing the paramount importance of transparency, enterprises should emphasize the disclosure of ESG issues. By improving the construction of the ESG information disclosure system, proactively assessing the quality of ESG information disclosure to regulatory authorities such as the China Securities Regulatory Commission (CSRC), and signaling a commitment to sustainable development, companies can cultivate trust among stakeholders, thereby laying a solid foundation for realizing long-term value. However, it is also necessary to guard against issues such as excessive public opinion guidance or overheating of investor sentiment. Therefore, relevant departments need to monitor public opinions and capital market sentiment in real-time to promote the rational investment of green funds.

Lastly, companies must embrace a pursuit of green innovation as a key driver for transformative change. This involves a robust commitment to intensifying R&D efforts geared towards the creation of green products and technologies. Such dedication serves to elevate a company's green reputation, making it an attractive prospect for investment from green funds. Specifically, companies should align their ESG governance practices with the preferences of green funds, actively introduce outstanding green innovative talents at home and abroad, and spearhead the large-scale adoption of green technology. Ultimately, these concerted efforts will contribute to healthy and sustainable corporate growth. However, enterprises should also regularly assess green innovative talents, and adjust innovation direction according to the forefront of green knowledge and technology to ensure the effectiveness of their own ESG construction.

*Reference*

Ma W, Duan X, Tang Y. Enterprise sustainable development and green fund concern: The analysis and test of R&D from listed companies in China. Energy Economics. 2023;121:106654. doi: 10.1016/j.eneco.2023.106654

He F, Yan Y, Hao J, Wu J (George). Retail investor attention and corporate green innovation: Evidence from China. Energy Economics. 2022;115:106308. doi: 10.1016/j.eneco.2022.106308

Naqvi B, Mirza N, Rizvi SKA, Porada-Rochoń M, Itani R. Is there a green fund premium? Evidence from twenty seven emerging markets. Global Finance Journal. 2021;50:100656. doi: 10.1016/j.gfj.2021.100656

Ji X, Zhang Y, Mirza N, Umar M, Rizvi SKA. The impact of carbon neutrality on the investment performance: Evidence from the equity mutual funds in BRICS. Journal of Environmental Management. 2021;297:113228. doi: 10.1016/j.jenvman.2021.113228

Jiang L, Bai Y. Strategic or substantive innovation? -The impact of institutional investors’ site visits on green innovation evidence from China. Technology in Society. 2022;68:101904. doi: 10.1016/j.techsoc.2022.101904

Siemroth C, Hornuf L. Why Do Retail Investors Pick Green Investments? A Lab-in-the-Field Experiment with Crowdfunders. Journal of Economic Behavior & Organization. 2023;209:74–90. doi: 10.1016/j.jebo.2023.02.023

Goodell JW, Corbet S, Yadav MP, Kumar S, Sharma S, Malik K. Time and frequency connectedness of green equity indices: Uncovering a socially important link to Bitcoin. International Review of Financial Analysis. 2022;84:102379. doi: 10.1016/j.irfa.2022.102379

Sangiorgi I, Schopohl L. Why do institutional investors buy green bonds: Evidence from a survey of European asset managers. International Review of Financial Analysis. 2021;75:101738. doi: 10.1016/j.irfa.2021.101738

Chatnani NN. Green Investing and Indian Investors: The Case of Suzlon Energy. FIIB Business Review. 2018;7(1):16–21. doi: 10.1177/2319714518763395

Silva F, Cortez MC. The performance of US and European green funds in different market conditions. Journal of Cleaner Production. 2016;135:558–66. doi: 10.1016/j.jclepro.2016.06.112

**Reply to Reviewer 2**

Dear Professor,

We sincerely thank you for your helpful and constructive comments and suggestions. We have considered your advice very seriously and incorporated it into the revised paper. We believe the paper is much improved and sincerely thank you for the advice and encouragement. Thank you for your kind comments on this manuscript. This statement details the changes we made, and it is keyed to your report. For ease of reading, we have included the original comments in bold font, followed by our detailed response in highlighted blue font. The reply to the comments is listed as follows.

**The paper examines the effects of green funds and green innovation on ESG performance of publicly listed Chinese companies from 2010 to 2021. The research findings suggest that green funds can positively affect corporate ESG performance. Mechanism analysis indicates that green funds contribute to elevated ESG performance by mitigating financial constraints, enhancing managerial efficiency, and fostering green innovation. Heterogeneity analysis accentuates that the effect of green funds is particularly potent in companies with high external attention. Furthermore, the engagement of green funds also exerts a significant affirmative influence on its production capabilities and economic value. The paper is interesting, and I have some comments for the authors to improve the paper.**

**[Comment 1] The sample period includes the COVID-19 lockdown period, which might cause some biases. The authors should control or isolate the potential impact of COVID-19 on their results.**

*Thanks for your comments. We have addressed this concern by conducting robustness tests, excluding samples from 2020 to 2021. The results remain consistent with our original findings. The revised manuscript is as follows:*

Since the substantial impact of COVID-19 on both capital market and corporate decision-making, we exclude the samples from 2020 to 2021. The regression results in column (1) of Table 8 reveal that the estimated coefficient of *lngreen* remains significantly positive.

**Table 8. Considering other important factors**

|  | (1) | (2) | (3) | (4) | (5) |
| --- | --- | --- | --- | --- | --- |
|  | Exclude the impact of COVID-19 | Control for environmental regulations | Exclude abnormal information disclosure  samples | Keep high-quality information disclosure samples | Control for greenwashing of companies |
|  | *ESG* | *ESG* | *ESG* | *ESG* | *ESG* |
| *lngreen* | 0.0598^***^ | 0.0652^***^ | 0.0596*** | 0.0522*** | 0.0641*** |
|  | (0.0116) | (0.0108) | (0.0111) | (0.0106) | (0.0109) |
| *LCCP* |  | 0.0789^**^ |  |  |  |
|  |  | (0.0305) |  |  |  |
| *Carbon* |  | 0.0211 |  |  |  |
|  |  | (0.0317) |  |  |  |
| *GCP* |  | 0.0302 |  |  |  |
|  |  | (0.0414) |  |  |  |
| *gws* |  |  |  |  | -0.0006 |
|  |  |  |  |  | (0.0250) |
| Controls | Yes | Yes | Yes | Yes | Yes |
| Firm fixed effects | Yes | Yes | Yes | Yes | Yes |
| Year fixed effects | Yes | Yes | Yes | Yes | Yes |
| N | 23307 | 29991 | 24691 | 26640 | 29449 |
| Adj. R^2^ | 0.5043 | 0.5132 | 0.5316 | 0.5174 | 0.5160 |

**[Comment 2] It will also be interesting for the readers to see how the effects of green funds on ESG performance may vary due to national and international environmental regulations such as the Paris Climate Agreement.**

**Response:**

*Thanks for your comments. In the robustness test, we take into account the overall impact of environmental regulations. We also consider the separate impact of each environmental regulation. The conclusions remain robust. The revised manuscript is as follows:*

Additionally, we further account for the potential influence of environmental regulations during the sample period by introducing low-carbon city pilot policy (*LCCP*), carbon market trading pilot policy (*Carbon*), and green credit policy (*GCP*) into the regression model. According to the estimation results in column (2) of Table 8, the fundamental conclusions of the study remain robust.

**Table 8. Considering other important factors**

|  | (1) | (2) | (3) | (4) | (5) |
| --- | --- | --- | --- | --- | --- |
|  | Exclude the impact of COVID-19 | Control for environmental regulations | Exclude abnormal information disclosure  samples | Keep high-quality information disclosure samples | Control for greenwashing of companies |
|  | *ESG* | *ESG* | *ESG* | *ESG* | *ESG* |
| *lngreen* | 0.0598^***^ | 0.0652^***^ | 0.0596*** | 0.0522*** | 0.0641*** |
|  | (0.0116) | (0.0108) | (0.0111) | (0.0106) | (0.0109) |
| *LCCP* |  | 0.0789^**^ |  |  |  |
|  |  | (0.0305) |  |  |  |
| *Carbon* |  | 0.0211 |  |  |  |
|  |  | (0.0317) |  |  |  |
| *GCP* |  | 0.0302 |  |  |  |
|  |  | (0.0414) |  |  |  |
| *gws* |  |  |  |  | -0.0006 |
|  |  |  |  |  | (0.0250) |
| Controls | Yes | Yes | Yes | Yes | Yes |
| Firm fixed effects | Yes | Yes | Yes | Yes | Yes |
| Year fixed effects | Yes | Yes | Yes | Yes | Yes |
| N | 23307 | 29991 | 24691 | 26640 | 29449 |
| Adj. R^2^ | 0.5043 | 0.5132 | 0.5316 | 0.5174 | 0.5160 |

**Table R1. Considering the impact of environmental regulations**

|  | (1) | (2) | (3) |
| --- | --- | --- | --- |
|  | *ESG* | *ESG* | *ESG* |
| *lngreen* | 0.0653^***^ | 0.0653^***^ | 0.0656^***^ |
|  | (0.0108) | (0.0108) | (0.0108) |
| *LCCP* | 0.0792^***^ |  |  |
|  | (0.0304) |  |  |
| *Carbon* |  | 0.0237 |  |
|  |  | (0.0315) |  |
| *GCP* |  |  | 0.0242 |
|  |  |  | (0.0413) |
| Controls | Yes | Yes | Yes |
| Firm fixed effects | Yes | Yes | Yes |
| Year fixed effects | Yes | Yes | Yes |
| N | 29991 | 29991 | 29991 |
| Adj. R^2^ | 0.5132 | 0.5130 | 0.5130 |

*We also conduct regressions grouped by regional environmental regulatory intensity to examine variations in the effectiveness under heterogeneous environmental regulatory conditions. The revised manuscript is as follows:*

Government environmental regulations are crucial in curbing environmental degradation and driving enterprises towards green development. By employing incentive-based and mandatory measures, government-led environmental regulatory approaches complement the external oversight exerted by green funds on corporate environmental and social performance. The complementary effect between green funds and government environmental regulations suggests that the impact of green funds on corporate ESG performance enhancement is more substantial in regions with weaker environmental regulations. To assess the level of government environmental regulatory intensity faced by companies, we select relevant vocabulary terms that comprehensively reflect the intensity of government environmental regulations. These terms are then analyzed using word frequency statistics from municipal government work reports to assess the level of government environmental regulatory intensity faced by companies. In columns (1) and (2) of Table 19, the results indicate a higher coefficient of *lngreen* in the weaker environment regulatory group, showing that the effect of green funds on corporate ESG improvement is more potent in regions with less stringent environmental regulations.

**Table 19. Heterogeneous analysis: region characteristics**

|  | (1) | (2) | (3) | (4) | (5) | (6) |
| --- | --- | --- | --- | --- | --- | --- |
|  | Environmental regulation | | Air pollution | | Marketability | |
|  | Low | High | Low | High | Low | High |
| *lngreen* | 0.0777^***^ | 0.0516^***^ | 0.0473^***^ | 0.0660^***^ | 0.0795^***^ | 0.0619^***^ |
|  | (0.0160) | (0.0141) | (0.0175) | (0.0138) | (0.0280) | (0.0118) |
| Controls | Yes | Yes | Yes | Yes | Yes | Yes |
| Firm fixed effects | Yes | Yes | Yes | Yes | Yes | Yes |
| Year fixed effects | Yes | Yes | Yes | Yes | Yes | Yes |
| N | 14964 | 15027 | 13705 | 16286 | 4440 | 25551 |
| Adj. R^2^ | 0.5454 | 0.5154 | 0.5945 | 0.5098 | 0.5070 | 0.5120 |
| Experience P-value | 0.007^***^ | | 0.021^**^ | | 0.000^***^ | |

**[Comment 3] The authors need to clarify how they control for greenwashing when they identify green funds. This is crucial as many funds may simply use environmentally relevant terms to promote their funds without actually being green (i.e. greenwashing).**

**Response:**

*Thank you for your valuable feedback. In addressing the potential issue of "greenwashing," our efforts involve several measures. Firstly, we have demonstrated that green funds exhibit a preference for entering clean industries and firms with superior environmental performance. The revised manuscript is as follows:*

**4.1 Validity testing of the selected indicators**

Since this research identifies the business goals and scope of investment funds combined with textual analysis, there is a real possibility that there might be the potential risk of “greenwashing” and other issues. Therefore, before conducting regression analysis, this study adopts various testing measures to verify the validity of selected indicators. Initially, we categorize the sample companies into polluting and clean industries based on their industrial attributes, grouping them according to the median of environmental performance indicators from the Huazheng ESG Rating. Subsequently, we observe the entry of green funds, presenting the results in Table 2 to demonstrate that the mean values of both the number of green funds (*greennumber*) and the proportion of market value held by green funds to the net value of the company (*greenratio*) are significantly higher in clean industries and firms with better environmental performance. It initially suggests that green funds are more inclined to enter clean industries and firms with better environmental performance.

**Table 2. Descriptive statistics of distinguishing among different categorized enterprises**

| Classification Criteria |  | *greennumber* | | | *greenratio* | | |
| --- | --- | --- | --- | --- | --- | --- | --- |
|  | N | Mean | Min | Max | Mean | Min | Max |
| *Polluting Industry* | 7179 | 1.2999 | 0 | 50 | 0.0437 | 0 | 0.9819 |
| *Clean Industry* | 22812 | 1.6937 | 0 | 68 | 0.0644 | 0 | 0.9819 |
| Mean difference test (t-value) |  | 8.7055*** | | | 9.7925*** | | |
| *Better Environmental Performance Enterprises* | 15028 | 1.9327 | 0 | 68 | 0.0706 | 0 | 0.9819 |
| *Poor Environmental Performance Enterprises* | 14963 | 1.2647 | 0 | 48 | 0.0483 | 0 | 0.9819 |
| Mean difference test (t-value) |  | 17.3720*** | | | 12.3743*** | | |

Furthermore, we argue our analysis by merging environmental information disclosure (*EID*) data of Chinese listed companies with our original database and examining whether the EID of companies attracts green funds to invest for further mitigating the potential risk of greenwashing. Specifically, EID contains eight dimensions of environmental information disclosure of listed companies, including environmental protection concept (EPC), environmental protection goal (EG), environmental management system (EMS), environmental protection education and training (EDT), environmental protection unique action (EA), environmental emergency mechanism (EEM), environmental protection honors or rewards (EHA), and three simultaneous systems (TS). According to the regression results in Table 3, the high-quality environmental information disclosure of enterprises improves the investment willingness of green funds, which indicates that the investment field of green funds also attaches great importance to the disclosure of the enterprise's environmental protection concept, environmental management system construction, environmental protection particular action and other essential measures. It indirectly confirms that the green funds we identified have a close relationship with the environmental protection behaviors of the enterprises. The analysis above confirms that green funds flow to enterprises and projects with green attributes and high-quality environmental information disclosure indeed, which reduces the possibility of "greenwashing."

**Table 3. Corporate EID and entry of green funds**

|  | (1) | (2) | (3) | (4) | (5) | (6) | (7) | (8) |
| --- | --- | --- | --- | --- | --- | --- | --- | --- |
|  | *EPC* | *EG* | *EMS* | *EDT* | *EA* | *EEM* | *EHA* | *TS* |
|  | *lngreen* | *lngreen* | *lngreen* | *lngreen* | *lngreen* | *lngreen* | *lngreen* | *lngreen* |
| *EID* | 0.0314^***^ | 0.0180 | 0.0175^*^ | 0.0307^**^ | 0.0459^***^ | 0.0506^***^ | 0.0371^***^ | 0.0700^***^ |
|  | (0.0113) | (0.0143) | (0.0105) | (0.0156) | (0.0138) | (0.0139) | (0.0136) | (0.0190) |
| Controls | Yes | Yes | Yes | Yes | Yes | Yes | Yes | Yes |
| Firm fixed effects | Yes | Yes | Yes | Yes | Yes | Yes | Yes | Yes |
| Year fixed effects | Yes | Yes | Yes | Yes | Yes | Yes | Yes | Yes |
| N | 29991 | 29991 | 29991 | 29991 | 29991 | 29991 | 29991 | 29991 |
| Adj. R^2^ | 0.5353 | 0.5352 | 0.5352 | 0.5352 | 0.5354 | 0.5355 | 0.5353 | 0.5356 |

*Secondly, we have excluded samples with abnormal information disclosure and kept high-quality information disclosure samples. The revised manuscript is as follows:*

In light of the greenwashing phenomenon, we conduct a series of rigorous tests. Companies penalized by CSRC or stock exchanges during the sample period, notably due to issues like information disclosure, are excluded. The baseline results, as depicted in Column (3) of Table 8, retain their validity. What’s more, our focus narrow to listed companies with excellent or good information disclosure evaluation results from the Shenzhen and Shanghai Stock Exchange. In Column (4) of Table 8, the baseline results persist.

*Lastly, we have incorporated corporate greenwashing as a control variable, and the conclusions drawn from these methodologies remain robust.*

Following Hu et al.(2023) [66], we effectively control for companies' greenwashing behavior in the baseline regression. As illustrated in Column (5) of Table 8, the baseline results endure.

**Table 8. Considering other important factors**

|  | (1) | (2) | (3) | (4) | (5) |
| --- | --- | --- | --- | --- | --- |
|  | Exclude the impact of COVID-19 | Control for environmental regulations | Exclude abnormal information disclosure  samples | Keep high-quality information disclosure samples | Control for greenwashing of companies |
|  | *ESG* | *ESG* | *ESG* | *ESG* | *ESG* |
| *lngreen* | 0.0598^***^ | 0.0652^***^ | 0.0596*** | 0.0522*** | 0.0641*** |
|  | (0.0116) | (0.0108) | (0.0111) | (0.0106) | (0.0109) |
| *LCCP* |  | 0.0789^**^ |  |  |  |
|  |  | (0.0305) |  |  |  |
| *Carbon* |  | 0.0211 |  |  |  |
|  |  | (0.0317) |  |  |  |
| *GCP* |  | 0.0302 |  |  |  |
|  |  | (0.0414) |  |  |  |
| *gws* |  |  |  |  | -0.0006 |
|  |  |  |  |  | (0.0250) |
| Controls | Yes | Yes | Yes | Yes | Yes |
| Firm fixed effects | Yes | Yes | Yes | Yes | Yes |
| Year fixed effects | Yes | Yes | Yes | Yes | Yes |
| N | 23307 | 29991 | 24691 | 26640 | 29449 |
| Adj. R^2^ | 0.5043 | 0.5132 | 0.5316 | 0.5174 | 0.5160 |

**[Comment 4] The results must be discussed in light of the current literature to augment the contributions of the paper.**

**Response:**

*Thank you for your constructive feedback. We appreciate your guidance on discussing the results in the context of the current literature to strengthen the contributions of our paper. In response to your suggestion, we have revised the manuscript to provide a more detailed discussion of how our study contributes to the existing literature.*

*Our study makes significant contributions to the literature in three aspects. Firstly,* *we expand the research on the determinants of corporate ESG performance. This expansion of the research scope enhances our understanding of the multifaceted factors influencing corporate sustainable development. Secondly, while there is a substantial body of literature exploring the general impact of green finance on corporate performance, our study adds value by specifically examining the nuanced effects of green funds on corporate ESG performance. This focused exploration deepens our understanding of the intricate relationship between green finance and sustainable development. Lastly, our research explores the heterogeneous effects of green funds on corporate ESG performance from a micro-level perspective. It broadens the analysis to encompass how external scrutiny impacts corporate ESG performance.*

*The text in the introduction section of the revised draft is as follows:*

The study generates several potential contributions. Firstly, it expands the research on the determinants of corporate ESG performance. While previous scholars have explored some factors influencing corporate ESG performance from the perspective of digital financial development [17], digital technological innovation [18], corporate digital transformation [19], managerial attributes [20,21], corporate governance structures [22,23], and government environmental attention [24–26], this study aims to investigate the impact of green funds on corporate ESG performance and add to the factors contributing to corporate ESG performance. Furthermore, it reveals how green funds impact corporate ESG performance through three channels: financial constraints, managerial efficiency, and green innovation. Our study not only adds to the theoretical understanding of corporate sustainable development but also offers practical insights that can inform strategies for fostering ESG performance.

Secondly, a substantial body of literature has widely discussed the effect of green finance on corporate performance. Green finance offers various financial services for firms engaged in sustainable green projects and exerts external constraints to regulate the use of green funds and the environmental protection behavior of enterprises [3]. As for economic performance, prior studies discovered that green finance can lower debt financing costs [27], increase R&D investments [28], inhibit corporate over-investment behavior [29], enhance total factor productivity [30,31], and bolster market value [32]. As for environmental performance, green finance can stimulate green innovation [33,34], deter greenwashing behaviors [3], reduce energy consumption intensity [35], curtail carbon emissions [36], and facilitate the green transformation of heavily polluting enterprises [37,38]. However, the previous research mainly investigated the effects of green loans and bonds on micro-level corporate behavior. As an essential branch of green finance, green funds are bound to positively impact enterprises’ sustainable development. To overcome these listed situations, this article launches an experimental exploration of how green funds impact corporate ESG performance. The results would better prompt enterprises to recognize the significance of green fund investments.

Lastly, this study considers the heterogeneous effects of green funds on corporate ESG performance from a micro-level perspective, broadening the analysis to encompass how external scrutiny impacts corporate ESG performance. External attention is primarily characterized by external pressures rather than incentives [39]. The study reveals notable variations in the influence of external attention from media, analysts, and the general public [39–41], extending the body of research on how external examination influences corporate ESG practices and providing valuable evidence on how green funds effectively guide companies to enhance their ESG performance.

*What’s more, in the discussion section at the end of the article, we introduced supplementary comparisons of similar research scenarios from domestic and foreign literature to highlight the contributions of this article, The specific ideas are mainly:*

Although this study only uses listed companies in China as the research sample, the conclusions are practically relevant for many countries with emerging economies, including China, for two main reasons. Firstly, most emerging economies share similar characteristics with China since their green financial systems are not yet fully developed and the concept of green sustainable development is yet to be promoted [75,76]. Therefore, exploring how to guide sustainable development with the support of green funds is an integral part of green financial reform. Secondly, literature about green funds in different regions also provides support for the applicability of our conclusions. Ma et al. (2023) [61] discussed the relationship among green fund concerns, corporate R&D investment and sustainable development. He et al. (2022) [69] and Jiang and Bai (2022) [77] analyzed the differential impact of institutional investors on corporate green innovation. In addition, Siemroth and Hornuf (2023) [78] found that investors prefer positive environmental impact and drive corporate transformation by investing in green projects. As for the performance of green funds in international markets, Goodell et al. (2022) [79] discovered that green funds are related to the development of financial technology. Ji et al. (2021) [76] revealed that the green funds of BRICS have performed better than other types of funds. Sangiorgi and Schopohl (2021) [80] and Chatnani (2018) [81] also found green investing funds in Europe and India have a better performance in stock price. Silva and Cortez (2016) [82] found that the performance of US and European green funds is higher in crisis periods. In general, our study is based on the above research and further focuses on the impact of green funds on the sustainable development of enterprises, rather than the single dimension of corporate environmental performance, stock price, or investment return considered in previous literature.

Our study extends the aforementioned research and delves deeper into examining green funds and corporate ESG performance. However, it is essential to note that this paper has yet to explicitly address the impact of small and medium-sized enterprises (SMEs), which remain pivotal to economic development in many emerging economies. Therefore, future research could be broadened to include SME data.

**[Comment 5] The paper requires proofreading to check for grammatical and spelling errors.**

**Response:**

*We apologize for these types in the original manuscript. We have corrected them as you suggested. In addition, we have also carefully checked and corrected potential errors related to grammar, syntax, consistency, and language choice. Moreover, we have used a professional editorial service to help us polish the flow, style, and language in this revised manuscript.*

*Reference*

Zhang D. Does green finance really inhibit extreme hypocritical ESG risk? A greenwashing perspective exploration. Energy Economics. 2023;121:106688. doi: 10.1016/j.eneco.2023.106688

Ren X, Zeng G, Zhao Y. Digital finance and corporate ESG performance: Empirical evidence from listed companies in China. Pacific-Basin Finance Journal. 2023;79:102019. doi: 10.1016/j.pacfin.2023.102019

Huang Q, Fang J, Xue X, Gao H. Does digital innovation cause better ESG performance? an empirical test of a-listed firms in China. Research in International Business and Finance. 2023;66:102049. doi: 10.1016/j.ribaf.2023.102049

Fang M, Nie H, Shen X. Can enterprise digitization improve ESG performance? Economic Modelling. 2023;118:106101. doi: 10.1016/j.econmod.2022.106101

Aabo T, Giorici IC. Do female CEOs matter for ESG scores? Global Finance Journal. 2023;56:100722. doi: 10.1016/j.gfj.2022.100722

Welch K, Yoon A. Do high-ability managers choose ESG projects that create shareholder value? Evidence from employee opinions. Rev Account Stud. 2022; doi: 10.1007/s11142-022-09701-4

Wang L, Qi J, Zhuang H. Monitoring or Collusion? Multiple Large Shareholders and Corporate ESG Performance: Evidence from China. Finance Research Letters. 2023;53:103673. doi: 10.1016/j.frl.2023.103673

Tian Z, Zhu B, Lu Y. The governance of non-state shareholders and corporate ESG: Empirical evidence from China. Finance Research Letters. 2023;56:104162. doi: 10.1016/j.frl.2023.104162

Shu H, Tan W. Does carbon control policy risk affect corporate ESG performance? Economic Modelling. 2023;120:106148. doi: 10.1016/j.econmod.2022.106148

Zhang D, Meng L, Zhang J. Environmental subsidy disruption, skill premiums and ESG performance. International Review of Financial Analysis. 2023;90:102862. doi: 10.1016/j.irfa.2023.102862

Wang H. Does the low-carbon transition promote the ESG disclosure of renewable energy enterprises? Environ Sci Pollut Res. 2023; doi: 10.1007/s11356-023-28788-z

Shi J, Yu C, Li Y, Wang T. Does green financial policy affect debt-financing cost of heavy-polluting enterprises? An empirical evidence based on Chinese pilot zones for green finance reform and innovations. Technological Forecasting and Social Change. 2022;179:121678. doi: 10.1016/j.techfore.2022.121678

Zhang D, Kong Q. Credit policy, uncertainty, and firm R&D investment: A quasi-natural experiment based on the Green Credit Guidelines. Pacific-Basin Finance Journal. 2022;73:101751. doi: 10.1016/j.pacfin.2022.101751

Yan C, Mao Z, Ho KC. Effect of green financial reform and innovation pilot zones on corporate investment efficiency. Energy Economics. 2022;113:106185. doi: 10.1016/j.eneco.2022.106185

Cui X, Wang P, Sensoy A, Nguyen DK, Pan Y. Green Credit Policy and Corporate Productivity: Evidence from a Quasi-natural Experiment in China. Technological Forecasting and Social Change. 2022;177:121516. doi: 10.1016/j.techfore.2022.121516

Li Y, Yu C, Shi J, Liu Y. How does green bond issuance affect total factor productivity? Evidence from Chinese listed enterprises. Energy Economics. 2023;106755. doi: 10.1016/j.eneco.2023.106755

Khurram MU, Xie W, Mirza SS, Tong H. Green bonds issuance, innovation performance, and corporate value: Empirical evidence from China. Heliyon. 2023;9(4):e14895. doi: 10.1016/j.heliyon.2023.e14895

Chen Z, Zhang Y, Wang H, Ouyang X, Xie Y. Can green credit policy promote low-carbon technology innovation? Journal of Cleaner Production. 2022;359:132061. doi: 10.1016/j.jclepro.2022.132061

Liu S, Wang Y. Green innovation effect of pilot zones for green finance reform: Evidence of quasi natural experiment. Technological Forecasting and Social Change. 2023;186:122079. doi: 10.1016/j.techfore.2022.122079

Su Z, Guo Q, Lee HT. Green finance policy and enterprise energy consumption intensity: Evidence from a quasi-natural experiment in China. Energy Economics. 2022;115:106374. doi: 10.1016/j.eneco.2022.106374

Zhang A, Deng R, Wu Y. Does the green credit policy reduce the carbon emission intensity of heavily polluting industries? -Evidence from China’s industrial sectors. Journal of Environmental Management. 2022;311:114815. doi: 10.1016/j.jenvman.2022.114815

Li R, Chen Y. The influence of a green credit policy on the transformation and upgrading of heavily polluting enterprises: A diversification perspective. Economic Analysis and Policy. 2022;74:539–52. doi: 10.1016/j.eap.2022.03.009

Lu Y, Gao Y, Zhang Y, Wang J. Can the green finance policy force the green transformation of high-polluting enterprises? A quasi-natural experiment based on “Green Credit Guidelines.” Energy Economics. 2022;114:106265. doi: 10.1016/j.eneco.2022.106265

Geng Y, Chen J, Liu T, Tao D. Public environmental attention, media coverage, and corporate green innovation: evidence from heavily polluting industries in China. Environ Sci Pollut Res. 2023;30(37):86911–26. doi: 10.1007/s11356-023-28369-0

Gu Y, Ho KC, Yan C, Gozgor G. Public environmental concern, CEO turnover, and green investment: Evidence from a quasi-natural experiment in China. Energy Economics. 2021;100:105379. doi: 10.1016/j.eneco.2021.105379

Huang M, Li M, Liao Z. Do politically connected CEOs promote Chinese listed industrial firms’ green innovation? The mediating role of external governance environments. Journal of Cleaner Production. 2021;278:123634. doi: 10.1016/j.jclepro.2020.123634

Ma W, Duan X, Tang Y. Enterprise sustainable development and green fund concern: The analysis and test of R&D from listed companies in China. Energy Economics. 2023;121:106654. doi: 10.1016/j.eneco.2023.106654

Hu X, Hua R, Liu Q, Wang C. The green fog: Environmental rating disagreement and corporate greenwashing. Pacific-Basin Finance Journal. 2023;78:101952. doi: 10.1016/j.pacfin.2023.101952

He F, Yan Y, Hao J, Wu J (George). Retail investor attention and corporate green innovation: Evidence from China. Energy Economics. 2022;115:106308. doi: 10.1016/j.eneco.2022.106308

Naqvi B, Mirza N, Rizvi SKA, Porada-Rochoń M, Itani R. Is there a green fund premium? Evidence from twenty seven emerging markets. Global Finance Journal. 2021;50:100656. doi: 10.1016/j.gfj.2021.100656

Ji X, Zhang Y, Mirza N, Umar M, Rizvi SKA. The impact of carbon neutrality on the investment performance: Evidence from the equity mutual funds in BRICS. Journal of Environmental Management. 2021;297:113228. doi: 10.1016/j.jenvman.2021.113228

Jiang L, Bai Y. Strategic or substantive innovation? -The impact of institutional investors’ site visits on green innovation evidence from China. Technology in Society. 2022;68:101904. doi: 10.1016/j.techsoc.2022.101904

Siemroth C, Hornuf L. Why Do Retail Investors Pick Green Investments? A Lab-in-the-Field Experiment with Crowdfunders. Journal of Economic Behavior & Organization. 2023;209:74–90. doi: 10.1016/j.jebo.2023.02.023

Goodell JW, Corbet S, Yadav MP, Kumar S, Sharma S, Malik K. Time and frequency connectedness of green equity indices: Uncovering a socially important link to Bitcoin. International Review of Financial Analysis. 2022;84:102379. doi: 10.1016/j.irfa.2022.102379

Sangiorgi I, Schopohl L. Why do institutional investors buy green bonds: Evidence from a survey of European asset managers. International Review of Financial Analysis. 2021;75:101738. doi: 10.1016/j.irfa.2021.101738

Chatnani NN. Green Investing and Indian Investors: The Case of Suzlon Energy. FIIB Business Review. 2018;7(1):16–21. doi: 10.1177/2319714518763395

Silva F, Cortez MC. The performance of US and European green funds in different market conditions. Journal of Cleaner Production. 2016;135:558–66. doi: 10.1016/j.jclepro.2016.06.112

1. The green background of a fund manager encompasses both educational and professional aspects. Green-related education is determined based on whether their educational background falls within fields such as pulp and paper, environmental studies, environmental engineering, environmental science, and similar disciplines. Green work experience is assessed by examining their professional history or positions held, specifically looking for roles within environmental departments, environmental protection agencies, environmental committees, or individuals responsible for corporate pollution control.
