# Supplementary material for: Can green funds improve corporate environmental, social, and governance performance? Evidence from Chinese-listed companies
Source: PLoS One. 2024 Mar 28;19(3):e0301395. doi: 10.1371/journal.pone.0301395 (PMC10977774; doi:10.1371/journal.pone.0301395)
Supplement: Attachment — Submitted filename: Response+to+Reviewers.docx [file pone.0301395.s004.docx]

**Reply to Editors**

Dear editors,

We sincerely thank you for your helpful and constructive comments and suggestions. We have considered your advice very seriously and incorporated it into the revised paper. We believe the paper is much improved and sincerely thank you for the advice and encouragement. Thank you for your kind comments on this manuscript. This statement details the changes we made, and it is keyed to your report. For ease of reading, we have included the original comments in bold font, followed by our detailed response in highlighted blue font. The reply to the comments is listed as follows.

**[Comment 1] The control variables: Similar to the explanatory variable (in a robustness test), control variables should also be lagged by one period rather than at the same time point with the dependent variable. The authors may need to provide additional robustness test addressing this.**

**Response:**

*Thank you for your valuable suggestion. In response to your comment regarding the timing of control variables in our robustness tests, we have revised our analysis to include control variables that are lagged by one period. The revised manuscript is as follows:*

Recognizing that the effect of green funds on corporate ESG performance may extend beyond the current period, we further introduce a variable representing the number of green funds with a one-period lag (*L.lngreen*) into the regression model and lag all control variables by one-period (*L.Controls*). This extension allows us to examine the lagged effect of green funds on corporate ESG performance. The specific results are presented in Table 7. The coefficient of *L.lngreen* is significantly positive. However, the coefficient of *L.lngreen* (Column 2) is smaller than that of *lngreen* in the baseline model (Column 1). The lagged effect of green funds slightly decreases and remains significantly positive. It infers that the enhancement of ESG performance exhibits sustained positive considering lagged effect [66].

**Table 7. The lagged effect of green funds**

|  | (1) | (2) |
| --- | --- | --- |
|  | *ESG* | *ESG* |
| *lngreen* | 0.0655^***^ |  |
|  | (0.0108) |  |
| *L.lngreen* |  | 0.0463^***^ |
|  |  | (0.0117) |
| *Controls* | Yes | No |
| *L.Controls* | No | Yes |
| Firm fixed effects | Yes | Yes |
| Year fixed effects | Yes | Yes |
| N | 29,991 | 26,142 |
| Adj. R^2^ | 0.5130 | 0.5446 |

**[Comment 2] The Two-step Heckman selection estimation.**

**In this context, firms self-select themself into the control group or the treatment group, and such decision need to be addressed as it exhibits potential selection bias in the data. The use of the two-step Heckman selection model is reasonable. However, the Heckman selection model requires the modelling of the selection variables with at least one new variable that is not in the second stage. The modelling of the binary green fund choice in this revision includes the one-year lagged control variables, thus does not seem good determinants of the green fund choice. The authors also do not explain clearly the modelling of the green fund choice, and the factors that may determine the green fund choice. Factors that determine green fund choice may include financial constraints, the industry-average green fund choice (excluding the focal firm), and regulation pressure (may be measured by the climate policy uncertainty index for China by Lin and Zhao (2023)), then firm-level characteristics.**

**Lin, B. & Zhao, H. (2023). Tracking policy uncertainty under climate change. Resource Policy 83, 103699. https://doi.org/10.1016/j.resourpol.2023.103699**

**Response:**

*Thank you for your insightful comments regarding the two-step Heckman selection model in our study. We acknowledge the importance of addressing potential selection bias as firms self-select into the control or treatment group regarding green fund choices. In response to your feedback, we have revised our Heckman two-stage model analysis to include financial constraints, the industry-average green fund choice (excluding the focal firm), regulatory pressure, and other firm-level characteristics in our model. The revised manuscript is as follows:*

The tendency of green funds to opt for companies with outstanding operational performance and sound governance structures might result in self-selection issues within the analysis sample of this study. To mitigate the potential estimation bias arising from this concern, this study draws inspiration from the approach of He et al. [71] and employs the Heckman two-stage model for examination. The first-stage probit regression model is presented as follows:

$$green_{it}=\beta_{3}+\beta_{4}Controls_{t-1}+\beta_{5}SA_{t-1}+\beta_{6}Indgreenfund_{t-1}+\beta_{7}CPU_{t-1}+\varepsilon_{it-1}^{' ' '}$$

We use whether a company has green funds in the current year as the dependent variable. The independent variables include the SA index which reflects financial constraints(*SA*), the industry-average green fund shareholding proportion excluding the focal firm(*Indgreenfund*), the climate policy uncertainty index for China which reflects regulation pressure(*CPU*), and other corporate characteristics [72–74]. We lag these independent variables by one period because green funds rely on past corporate operational performance and macro conditions to decide whether to invest in a listed company.

Financial constraints limit the ability to allocate resources towards sustainability initiatives, which makes it hard for companies to take full advantage of green funds. Therefore, green funds tend to invest in enterprises with less financial constraints [73]. Industry-level green fund preferences reflect strict sectoral requirements regarding environmental responsibility. Enterprises in industries with strong green preferences must improve their environmental performance in order to gain competitive advantages and win the favor of green funds. Regulation pressure influences corporate environmental behaviors by imposing legal mandates, incentives, or penalties related to environmental compliance [72,74]. Therefore, enterprises accelerate green transformation to attract green funds. The business and governance conditions of companies are also important factors for the investment decisions of green fund investors. We calculate the corresponding inverse Mills ratio (*imr*) from the first-stage regression model and introduce it into the second-stage regression model. The second-stage regression model is presented as follows:

where the inverse Mills ratio is incorporated into the baseline regression as an additional control variable to rectify the potential sample self-selection bias in the baseline regression.

**Table 13. Heckman two-step estimations**

| (1) | | (2) | |
| --- | --- | --- | --- |
| First stage | | Second stage | |
| Variables | *green* | Variables | *ESG* |
| *L.Size* | 0.4400^***^ | *lngreen* | 0.0322^***^ |
|  | (0.0093) |  | (0.0115) |
| *L.Lev* | -0.3912^***^ | *Size* | 0.0724^***^ |
|  | (0.0539) |  | (0.0234) |
| *L.ROA* | 6.0426^***^ | *Lev* | -0.5052^***^ |
|  | (0.1868) |  | (0.0831) |
| *L.FirmAge* | -0.0862 | *ROA* | 0.9338^***^ |
|  | (0.0641) |  | (0.1422) |
| *L.Cashflow* | 1.0373^***^ | *FirmAge* | -0.3043^*^ |
|  | (0.1409) |  | (0.1748) |
| *L.TOP1* | -0.7969^***^ | *Cashflow* | -0.5198^***^ |
|  | (0.0599) |  | (0.1104) |
| *L.Indep* | 0.3064 | *TOP1* | 0.5826^***^ |
|  | (0.1900) |  | (0.1371) |
| *L.Board* | -0.1084^*^ | *Indep* | 1.4416^***^ |
|  | (0.0533) |  | (0.2447) |
| *L.SA* | -0.4575^***^ | *Board* | 0.0812 |
|  | (0.0889) |  | (0.0849) |
| *L.Indgreenfund* | 50.2583^***^ | *imr* | -0.5664^***^ |
|  | (2.6935) |  | (0.0250) |
| *L.CPU* | -0.0035^***^ |  |  |
|  | (0.0003) |  |  |
| Firm fixed effects | Yes |  | Yes |
| Year fixed effects | Yes |  | Yes |
| N | 26,142 |  | 26,142 |
| Adj. R^2^ |  |  | 0.5452 |

The two-stage regression results are presented in Table 13, revealing that the *imr* is significantly negative with sample selection issues. The core explanatory variable remains significantly positive after adjusting for the *imr*, indicating that the baseline regression results are robust after addressing sample selectivity concerns.

**Other comments:**

**[Comment 1] "14,874" instead of "14874". Please scan the whole paper to revise this number format.**

**Response:**

*Thank you for pointing out the formatting inconsistency. We have carefully scanned the entire document and revised all instances of number formatting to ensure consistency.*

**[Comment 2] The statement " on the other hand, the link between corporate ESG performance and the fund manager's previous green experience is fragile, satisfying the exogeneity of IV" (Section 4.4.1.) is exhausted. The authors need to revise this to provide a better rationale for why the IV is exogenous and satisfies the exclusion restrictions.**

**Response:**

*Thank you for your feedback. we have revised the instrumental variable section of our manuscript to provide a more comprehensive rationale for its exogeneity.* *The revised manuscript is as follows:*

On the other hand, the exogeneity of the instrument variable can be explained from two perspectives: Firstly, the green background of fund managers is usually driven by personal interests and future development goals, and is not directly related to corporate behaviors including ESG performance [70]. Secondly, the green background of fund managers is mostly determined in the early stage of their careers, and is not affected by the ESG performance of companies in the investment portfolio. This ensures that the green background is formed prior to investment decisions, thus satisfying the exclusion restrictions.

**[Comment 3] The definition of the *gws* variable is missing.**

**Response:**

*Thank you for your comments. We apologize for the omission of the definition for the greenwashing behavior variable (gws) in the original manuscript. We have revised our manuscript to include a definition of the greenwashing* *behavior variable(gws). The revised manuscript is as follows:*

Following Hu et al.(2023) [67], we effectively add corporate greenwashing behavior (*gws*) in the baseline regression. We examine corporate greenwashing behavior by comparing firms' verbal green claims with their actual environmental performance. Firstly, we create a list of words related to environmental topics. Then, we calculate the frequency of these words appearing in the Management Discussion and Analysis (MD&A) section of annual reports for each company. We create a dummy variable called *Oral*. If the frequency exceeds the industry median for that period, we label it as 1; otherwise, it is labeled as 0. Similarly, another dummy variable, *Actual*, is assigned a value of 1 if the company suffers environmental punishment during the year; otherwise, it is assigned a value of 0. Consequently, if *Oral*=1 and *Actual*=1, the corporate greenwashing behavior is set to 1; otherwise, it is set to 0.

*Reference*

Gan T, Li Y, Jiang Y. The impact of air pollution on venture capital: evidence from China. Environ Sci Pollut R. 2022;29(60):90615–31. doi: 10.1007/s11356-022-21972-7

Hu X, Hua R, Liu Q, Wang C. The green fog: Environmental rating disagreement and corporate greenwashing. Pacific-Basin Finance Journal. 2023;78:101952. doi: 10.1016/j.pacfin.2023.101952

Martin PR, Moser DV. Managers’ green investment disclosures and investors’ reaction. J Account Econ. 2016;61(1):239–54. doi: 10.1016/j.jacceco.2015.08.004

He F, Yan Y, Hao J, Wu J (George). Retail investor attention and corporate green innovation: Evidence from China. Energy Economics. 2022;115:106308. doi: 10.1016/j.eneco.2022.106308

Ren X, Zhang X, Yan C, Gozgor G. Climate policy uncertainty and firm-level total factor productivity: Evidence from China. Energ Econ. 2022;113:106209. doi: 10.1016/j.eneco.2022.106209

Cecere G, Corrocher N, Mancusi ML. Financial constraints and public funding of eco-innovation: empirical evidence from European SMEs. Small Bus Econ. 2020;54(1):285–302. doi: 10.1007/s11187-018-0090-9

Lin B, Zhao H. Tracking policy uncertainty under climate change. Resour Policy. 2023;83:103699. doi: 10.1016/j.resourpol.2023.103699

**Reply to Reviewers**

**[Reviewer1] Your research on the positive influence of green funds on corporate environmental, social, and governance (ESG) performance, as well as the underlying mechanisms driving this impact, provides valuable insights into sustainable development practices within the corporate sector. The systematic analysis of how green funds contribute to elevated ESG performance by alleviating financial constraints, enhancing managerial efficiency, and fostering green innovation is both thorough and compelling. Upon careful revision of your manuscript, I am assured that your research constitutes a substantial contribution to the academic field. I am of the opinion that the insights derived from your study will captivate our readership and foster a constructive dialogue within the realm of sustainable development and corporate sustainability.**

**Response:**

*Thank you for your encouraging feedback and constructive review of our manuscript. We are inspired by your supportive and insightful comments.* *We will strive for further research in the field of sustainable development.*

**[Reviewer2] Thank you for carefully addressing my comments. There is a minor point that can be addressed during the proof process. In Section 7, you should change the heading to Conclusion instead of Conclusion and Discussion.**

**Response:**

*Thank you for your comments. We change the heading to Conclusion in Section 7.*
